# Bead-Based Multiplex Assay for the Simultaneous Detection of Antibodies to African Swine Fever Virus and Classical Swine Fever Virus

**DOI:** 10.3389/fvets.2019.00306

**Published:** 2019-09-13

**Authors:** Cristina Aira, Tamara Ruiz, Linda Dixon, Sandra Blome, Paloma Rueda, Patricia Sastre

**Affiliations:** ^1^INGENASA, Inmunología y Genética Aplicada, Madrid, Spain; ^2^Virology Department, The Pirbright Institute, Pirbright, United Kingdom; ^3^Institute of Diagnostic Virology, Friedrich-Loeffler-Institute, Greifswald, Germany

**Keywords:** African swine fever, classical swine fever, multiplex, diagnosis, antibody

## Abstract

African swine fever (ASF) and Classical swine fever (CSF) are both highly contagious diseases of domestic pigs and wild boar. In the last years, several cases of both diseases have been reported in the Caucasus, Russian Federation and Eastern Europe. Thus, the probability of encountering these two viruses in the same area is increasing. Since differentiation by clinical or post-mortem examination is not possible, laboratory tools for differential diagnosis are required. In the present work, we have developed a triplex bead-based assay using some of the most immunogenic antigens of each virus, for the simultaneous detection of antibodies; i.e. the VP72 and VP30 of ASF virus (ASFV) and the E2 protein of CSF virus (CSFV). The assay was firstly set up and optimized using well characterized reference serum samples specific for each pathogen. Then, a panel of 352 sera from experimentally infected animals with either ASFV or CSFV were analyzed in the multiplex assay. A collection of 253 field negative sera was also included in the study. The results of the multiplex analysis were compared to those obtained by two commercially available ELISAs for detection of antibodies against ASFV or CSFV, and considered in this study as the reference techniques. The data obtained showed values of 97.3% sensitivity and 98.3% specificity for detection of antibodies to ASFV and 95.7% of sensitivity and 99.8% specificity for detection of antibodies to CSFV. This multiplex assay allows the simultaneous and differential detection of antibodies against ASFV and CSFV, providing a valuable tool for surveillance studies. Moreover, this method is rather versatile, offering the possibility of increasing the panel of antigens from other swine diseases that could be of interest for a differential diagnosis along with ASF and CSF.

## Introduction

African Swine Fever (ASF) is a highly infectious disease in swine population, caused by an enveloped double-stranded DNA virus, the ASF virus (ASFV), which is the only member of the Asfarviridae family (1). ASFV is composed of more than 68 structural proteins, many of which are highly immunogenic (2). Among them, the structural viral proteins (VP) VP72 and VP30 are commonly used for diagnostic purposes (3–5). ASFV infection causes a strong humoral immune response that persists for long periods of time, although neutralizing antibodies have consistently been described (6). There are no commercially available vaccines at the moment and therefore, the presence of antibodies in serum is a definitive indicator of infection. ASF control is based on early diagnosis and the enforcement of strict sanitary measures (7). Infection with ASFV correlates with a wide range of clinical syndromes from almost unapparent disease to a hemorrhagic fever with high fatality rates (95–100%) depending on the strain virulence and the immunological characteristics of the host (8, 9).

ASF was first described in Kenia in 1921 (10) and spread to other African, European, Caribbean and South American countries (11). The disease was successfully eradicated from all these territories, except for Sardinia and Sub-Saharan countries where the disease is still endemic (12). In 2007, ASF was introduced into Georgia, and since then it was spread into several Trans-Caucasian countries, the Russian Federation, Belarus, and Ukraine (13). Since 2007 to date, new outbreaks are continuously being reported in Eastern Europe and Russia (4, 14). During the last year, ASF has first been reported in China, Mongolia, Vietnam, Cambodia and spread to other countries in Asia is considered likely by the FAO (14, 15).

Classical Swine Fever (CSF) is also a highly contagious disease of pigs, caused by the CSF virus (CSFV), which is an enveloped singled-stranded, positive sense RNA virus belonging to the genus *Pestivirus* within the Flaviviridae family (16). CSFV has four structural proteins: the core protein (C) and three envelope glycoproteins: E1, E2, and E^rns^. E2 has been shown to be the most immunogenic protein of CSFV, inducing production of neutralizing antibodies and protection against lethal virus challenge (17, 18) what makes it a good candidate for diagnosis of CSF. CSFV infection presents different clinical manifestations which can vary from unapparent to peracute courses ending in the death of the animal, depending on virulence of the virus strain and host factors (19).

CSF was first reported in Ohio, USA in 1833 (20) and was widespread into Europe and America within a few years (21). After implementation of strict control measures, which include appropriate vaccination programs, several countries succeeded in eradicating CSF, including the United States, Australia and New Zealand; however, it continues to have a severe impact on Asia, Eastern Europe, and most of South and Central America as well as the Caribbean (22, 23). New outbreaks in the European Union keep occurring due to the viral introduction via wild boar, causing huge economic losses (14, 19, 24). Last year, CSF has also remerged in Japan and an ongoing case has been notified in the east coast of Russia (14, 25). This fact together with the spread of ASF from the Caucasus, increase the probability to encounter CSF and ASF in the same region and increase the necessity for fast differential diagnosis.

Since ASF and CSF cannot be differentiated by clinical nor post-mortem examination, laboratory tools for differential diagnosis of the two diseases are essential. Currently, there are some available tests for the simultaneous detection of ASF and CSF based on the direct detection by RT-PCR (26, 27) or in the indirect diagnosis by detection of specific antibodies by immunochromatography tests (28). These assays are of great value for immediate implementation of control measures to prevent further spread of the diseases.

A useful approach developed during the last decades for the multiplex diagnosis, are the bead-based multiplex assays (BBMAs). These are an alternative to planar microarrays, using colored code polystyrene microspheres as the solid support for the capture molecule, which are mixed in a single microtiter plate well to create a microarray in suspension. BBMAs reduce time, labor and sample volume requirements, allowing the testing of many samples for multiple targets simultaneously (29). The xMAP technology (Luminex) combines fluorescent-dyed microspheres, lasers, and digital signal processing up to 500 individual analytes within a single sample. This technology is widely applied in human health for different applications, such as strain identification in infections, immune response characterization (humoral and cellular), or biomarkers identification as well as other uses (30, 31). However, less work has been carried out using this technology in the veterinary field (32–38) and there are only a few commercial kits available. Moreover, when compared to conventional ELISA, previous results have shown that xMAP formats can be more sensitive and reproducible (35).

In this work, we have developed a triplex assay for detection of antibodies to ASFV and CSFV, using immunogenic antigens of each virus: VP72 and VP30 of ASFV and E2 of CSFV, as an approach for the simultaneous detection and differential diagnosis of both diseases. This approach could be a very useful tool in surveillance scenarios, preventing, or at least reducing, substantially economic losses to the swine industry.

## Materials and Methods

### Viral Antigens

The VP72 of ASFV was semi-purified by affinity chromatography with the monoclonal antibody 17LD3 (M.11.PPA.I17LD3; INGENASA, Madrid, Spain) from an inactivated extract of infected cells with ASFV strain (BA71). The VP30 of ASFV (BA71 strain) was produced with a 6X histidine tag in insect cells infected with a recombinant Baculovirus and further purified from the insoluble fraction under denaturing conditions. The glycoprotein E2 of CSFV (Brescia strain) was produced also in insect cells with a 6X histidine tag and purified from the culture media (secreted protein) by affinity chromatography with copper stabilized sepharose.

### Serum Samples

Reference serum for ASFV and CSFV, have been used for assay optimization. The ASFV-positive reference serum was provided by the European Union reference laboratory for ASF (EURL) and previously characterized by the OIE ELISA against the BA71 strain. The CSFV-positive reference serum was provided by the National and FAO reference laboratory for CSF at the Friedrich-Loeffler-Institut (FLI) and characterized by VNT (virus neutralization) against CSFV strain Alfort/187 with a 50% neutralization dose (ND50).

Two panels of well-characterized swine sera were included in the present study. For detection of antibodies to ASFV, a panel 333 serum samples from pigs used in vaccination/challenge experiments at BSL3 facilities at PIR, were included in this study. Briefly, 29 pigs were immunized with an attenuated Benin strain and serum samples were collected at 0, 2, 4, 7, 10, 15, 21, 28, 38, 43, 47, and 59 days post infection (dpi). The animals were boosted 21 days later with the same virus and on day 40 they were challenged with virulent Benin 97/1. A total of 115 samples were collected between 0 and 7 dpi, 57 samples between 8 and 15 dpi, 58 samples between 16 and 28 dpi and 103 samples taken after 1 month pi. (39). For detection of antibodies to CSFV, 30 experimental serum samples from pigs infected at FLI facilities were used (28). Briefly, 23 positive samples collected from pigs experimentally infected with the strain Alfort/187 of CSFV and 7 negative samples. Among these negative samples, one of them was an experimental negative sample and the other six were obtained from pigs infected with other serologically related *Pestivirus*: Border disease virus (BDV) and Bovine viral diarrhea virus (BVDV) (40). Finally, a collection of 253 negative field serum samples from Spanish farms free of both diseases were also evaluated.

In order to prepare pooled samples, each positive sample was spiked in negative serum to analyse a total of 5, 10, and 20 different sera per well. Negative sera were prepared by mixing equal volumes of 4, 9, and 19 negative field serum samples, respectively. This procedure was performed for one ASFV weak positive sample, one CSFV weak positive sample and a negative sample for both diseases. Pools were serially diluted in assay buffer, and the assay was performed as described for the triplex assay.

### Coupling of Target Antigens to Beads

The three viral target antigens were covalently coupled to different carboxylated magnetic bead regions (Luminexcorp, Austin, USA) with the xMAP® Antibody Coupling Kit following manufacturer's indications (ref. 40-50016, Luminexcorp, Austin, USA). Briefly, one million carboxylated magnetic microspheres, identified individually by a unique fluorescence ratio (regions #12, #15 and #25, MagPlex® Microspheres, Luminex) were activated according to the NHS/EDC protocol (41), based on a two-step carbodiimide reaction. Activated beads were incubated with different amounts of VP72, VP30, and E2, respectively, ranging from 2.5 to 10 μg per one million beads, in a final incubation volume of 500 μl, and incubated for 2 h with rotation in dark. After washing steps, supernatant was replaced with 1 ml of storage buffer (PBS, 1% BSA, 0.05% azide). Beads concentration after coupling was determined by counting on a Neubauer plate. The coupled microspheres were kept in storage buffer at 4°C in the dark until use, as recommended by manufacturer. The beads were used within the next 3 months after coupling.

A coupling confirmation assay was performed using serial dilutions of monoclonal specific antibodies to each protein: 18BG3 (INGENASA, Madrid, Spain) for VP72, anti-6X His tag (MA1-21315; Invitrogen, Carlsbad, CA) for VP30 and 14E11 (INGENASA, Madrid, Spain) for E2, in order to assess the coupling efficiency.

### Bead-Based Assay for Antibody Detection in Swine Serum

To perform the triplex assay, individual antigen-coupled microspheres were sonicated and vortexed for homogenization. A microsphere mixture was prepared mixing the three bead regions in assay buffer (PBS, 1% BSA) to a final concentration for each region of 25 beads/μl. Fifty microliters of this bead mixture was added over fifty microliters of individual pig serum samples diluted at 1/200 in assay buffer. Mixture was incubated for 1 h at room temperature (RT) and 650 rpm in a mini-shaker PSU-2T (Biosan). For this assay, 96-well plates (Stripwell^TM^ Microplate Medium binding Polystyrene, Costar) previously stabilized for 15 min, were used. The plate was protected from light during all the incubation process. After every incubation step, the plate was washed twice with washing buffer (PBS, 1% BSA, 0.05% Tween 20) using a magnetic washer. Each well was incubated with 50 μl of a polyclonal anti-swine antibody labeled with biotin (SAB3700436; Sigma-Aldrich, Kawasaki, Japan), at a final concentration of 4 μg/ml in assay buffer, for another hour in the same conditions. Then, 50 μl/well of Streptavidin R-phycoerythrin (Molecular probes®, life technologies) were added at a final concentration of 2 μg/ml in assay buffer and incubated for 30 min at the same conditions. The beads were then resuspended in washing buffer and the results were read out in a MAGPIX® dispositive (Luminex). The signal was measured as median fluorescence intensity (MFI) of at least, 50 events of each bead region.

Two wells per assay were incubated in absence of sample, only with assay buffer, as a blank signal, which is subtracted from the sample signal. Positive and negative controls were included in all assays to confirm the performance of the test.

### Statistical Analysis

Data were statistically analyzed by a ROC curve analysis using the MedCalc® 10 software to establish the optimal cut off value for each antigen.

For the statistical evaluation, samples were classified into positive or negative based on two commercial ELISAs which were used as the reference techniques in this study: INgezim 11.PPA.K3 for detection of specific antibodies against ASFV and INgezim 11.PPC.K3 for detection of specific antibodies against CSFV.

Statistical significance and 95% CI have been calculated for ASFV samples classified according to days post infection. For the statistical significance determination between ELISA and bead-based assay, a McNemar test has been performed.

## Results

### Development and Optimization of the Multiplex Bead-Based Assay

Optimal coupling amount was established as the minimum quantity of protein that gave a saturation signal of MFI in the titration curve. Thus, the following concentrations were used for each bead region: 10 μg of the VP72 (region #12), 5 μg of the VP30 (region #15), and 2.5 μg of the protein E2 (region #25) per one million beads.

**Figure 1:**
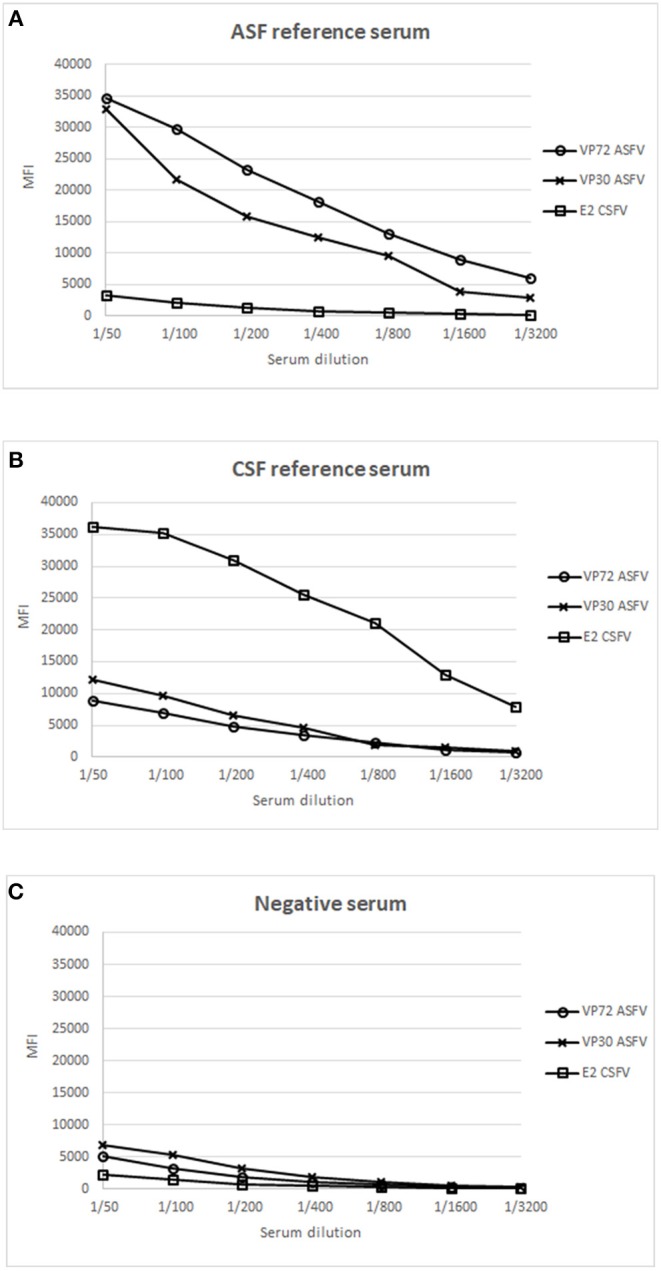
Establishment of optimal conditions for the development of a multiplex bead-based assay. X Axis shows the dilution value of the sera employed and Y Axis shows the Median Fluorescence Intensity (MFI). Response to different antigens is shown: (°) signal of bead #12 coupled to VP72, (x) signal of bead #15 coupled to VP30, and (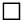) signal of bead #25 coupled to E2, using a reference serum for ASFV **(A)**, a reference serum for CSFV **(B)** and a negative serum for both diseases **(C)**.

Next, well-characterized reference swine serum samples for each pathogen were evaluated to establish the optimal conditions for screening purposes. Positive reference sera for ASFV and CSFV, respectively, and a serum from an animal free of both diseases, were included as positive and negative controls in this assay. Serial dilutions of each serum sample were incubated with the mix of the 3 bead regions and the assay was further performed as described in M&M. Figure 1A, shows the result of the ASF reference serum, giving a strong signal with VP72 and VP30, respectively, while no signal was detected against the E2, corresponding to the target antigen of CSFV. On the other hand, on Figure 1B, the reference serum for CSFV showed a strong signal with E2 antigen, while no significant reactivity with VP72 and VP30. Finally the negative serum showed no reactivity with neither of the antigens (Figure 1C). A 1/200 dilution of serum was selected as the optimal dilution for screening purposes. This dilution showed the highest responses to ASFV and CSFV antigens while no cross-reactivity to the non-target antigens in each case.

### Analysis of Experimental and Field Sera in the Multiplex Assay

Once the screening conditions were established, a collection of 605 swine sera were assessed in the triplex assay. A total of 333 experimental serum samples for ASF, 30 experimental serum samples for CSF and 253 field negative samples were included in the analysis. Out of the 333 experimental ASF sera, 185 were classified as positive by the 11.PPA.K3 and 11 as doubtful, so these were not included in the statistical analysis. Out of the 30 experimental sera for CSF, 23 were classified as positive by the 11.PPC.K3. The rest of the serum samples gave negative signals in both assays (Tables 1A,B).

**Table 1:** Sera characterization by INgezim 11.PPA.K3.

**Sample classification**	**Experimental ASF sera (PIR)**	**Experimental CSF sera (FLI)**	**Negative field samples**	**Total analyzed sera**
Positive	185	0	0	605
Negative	137	30	253	

**Table 1B:** Sera characterization by INgezim 11.PPC.K3.

**Sample classification**	**Experimental ASF sera (PIR)**	**Experimental CSF sera (FLI)**	**Negative field samples**	**Total analyzed sera**
Positive	0	23	0	605
Negative	322	7^†^	253	

† *Six out of these seven sera were obtained from animals infected with border disease virus (BDV) and Bovine viral diarrhea virus (BVDV), other Pestivirus serologically related to CSFV*.

**Figure 2:**
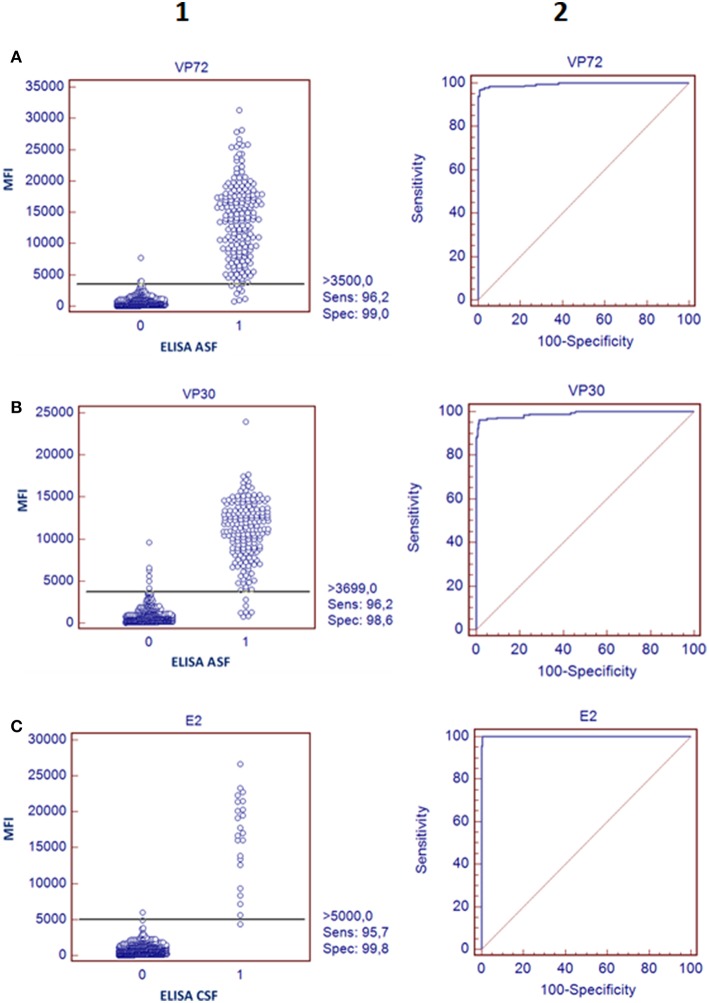
Validation of the bead-bead assay. The left panels represent a dot diagram where each dot represents an individual sample: results obtained for VP72 coupled to bead #12 **(A)** VP30 coupled to bead #15 **(B)**, and E2 antigen coupled to bead #25 **(C)**, The horizontal solid line corresponds to the cutoff values in each assay, according to the Medcalc software. X Axis shows the positive (1) or negative (0) classification of samples according to the ELISA used as reference technique in this study and Y Axis shows Median Fluorescence Intensity (MFI) obtained in the developed assay The right panels show a ROC curve analysis based on the data obtained in the bead-bead assay.

In regard to ASFV, a cut off value was established for each antigen according to the Medcalc software: 3500 and 3700 MFI for VP72 (#12) and VP30 (#15), respectively. With the developed assay, a sensitivity of the 96.2% for both antigens and a specificity of 99.0% and 98.6% for VP72 and VP30, respectively, were reached (Figures 2A,B). Particularly, more than 96% (178/185) of the samples classified as positive with the reference technique gave also a positive signal with the VP72 (bead #12) in the multiplex assay, and more than the 99% of the negative samples (416/420) gave also a negative signal in the developed multiplex assay. Four samples gave a false positive result when compared to the reference technique. Among these; three samples were obtained from sera at early days post-infection and the other sample corresponded to a positive serum to CSFV. Seven samples classified as positive with the reference technique, gave a negative signal for the VP72 antigen coupled to the region #12 (Table 2). For the detection of antibodies to the VP30 of ASFV similar results were obtained. More than 96% of the serum samples classified as positive by the reference technique were detected with the VP30 in the multiplex assay (178/185), and more than 98% of the serum samples classified as negative by the reference ELISA were also negative in the multiplex assay (414/420). Six samples that gave a negative result with the reference technique used in this study were positive to the bead #15 coupled to the VP30 antigen. All of these false positive samples were obtained at different days post-infection. Moreover, seven positive samples by the reference technique were not detected as positive in the multiplex assay (Table 2).

**Table 2:** Correlation between bead-based assay and the ELISAs used as reference for different antigens.

**No. of serum samples with ELISA**	**No. of serum samples with VP72 (bead #12)**			**No. of serum samples with VP30 (bead #15)**			**No. of serum samples with E2 (bead #25)**			**No. of serum samples with VP72 (#12) + VP30 (#15)**		
	**Pos**.	**Neg**.	**Total**	**Pos**.	**Neg**.	**Total**	**Pos**.	**Neg**.	**Total**	**Pos**.	**Neg**.	**Total**
Pos.	178	7	185	178	7	185	22	1	23	180	5	185
Neg.	4	416	420	6	414	420	1	581	582	7	413	420
Total	182	423	605	184	421	605	23	582	605	187	418	605
Sensitivity	96.2			96.2			95.7			97.3		
Specificity	99.0			98.6			99.8			98.3		

**Figure 3:**
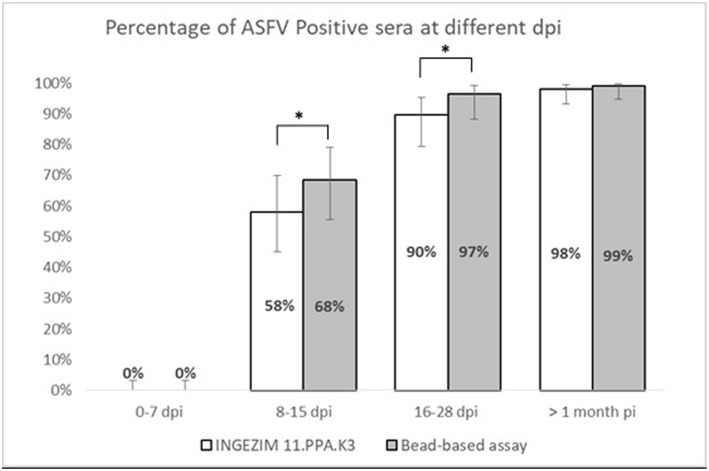
Stratified analysis of positive samples to ASFV according to days post-infection. X axis shows the percentage of positive samples within each group. Y axis shows different days post infection clustered as follows: 0–7 dpi, 8–15 dpi, 16–28 dpi, and > 1 month pi. Error bars show the 95% confidence interval for each bin of data. Statistical significance has been calculated according to a McNemar test, *p < 0.05.

Taking together the reactivity of a given serum against VP72 and VP30, the values of sensitivity and specificity were slightly increased (Table 2). More than the 97% (180/185) of the serum samples classified as positive were detected by, at least, one of the antigens. And more than the 98% (413/420) of the negative samples gave a negative signal to both antigens. By the combination of both antigens, only five false negative samples were obtained with the multiplex assay. The sensitivity parameter increased to 97.3% with a specificity of 98.3%. Additionally, a stratified analysis of the positive samples to ASFV according days post infection is shown in Figure 3. Within the first 7 dpi no positive results were observed in any of the techniques used. Between 8 and 15 dpi the bead-based assay gave a higher proportion of positive samples (68%) in the inoculated group than the technique used as reference (58%). The same observation was obtained in the 16–28 dpi group, in which bead-based assay exhibited 97% of positive samples, while a 90% was obtained with the reference technique. Samples collected a month after infection, gave similar results with both assays.

According to the cut-off value established by the ROC analysis (5000 MFI) for the E2 antigen (bead #25), the performance characteristics of the multiplex assay for CSF showed a good correlation with the reference technique, reaching a sensitivity of 95.7% and a specificity of 99.8% (Figure 2C). Negative samples for CSF, including disease-free animals and ASFV-infected pigs, gave clearly negative results showing no cross reactivity with the E2 antigen. The six serum samples obtained from animals infected with other related *Pestivirus* (BVDV or BDV), gave negative results in this assay format. Only one weak positive sample for CSF was not detected with the bead-based assay (Figure 2C, Table 2).

### Analysis of Pooled Samples for Surveillance Purposes

**Figure 4:**
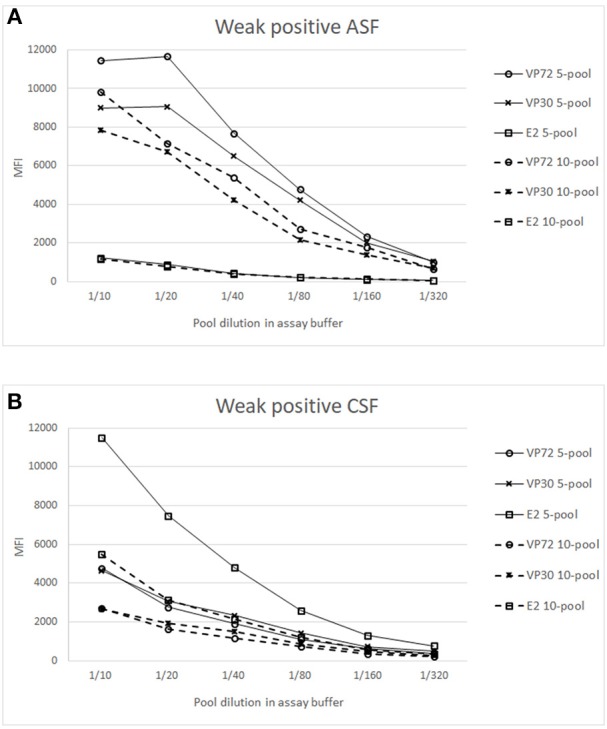
Analysis of weak positive samples in pooled conditions. X axis shows the dilution of the whole pool in assay buffer and Y axis the Median Fluorescence Intensity (MFI). Signals of weak positive sample for ASFV **(A)** or CSFV **(B)**, spiked in a 4 (**—**) or 9 (**- - -**) negative sera matrix are represented for (°) bead #12 coupled to VP72, (x) bead #15 coupled to VP30 and (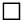) bead #25 coupled to E2. Cut off values for different antigens were established at 3500 (VP72), 3700 (VP30) and 5000 (E2) MFI.

To increase the high throughput screening possibilities of the assay, the capacity of analyzing samples from up to 20 animals per well was analyzed as described in M&M. Figure 4 shows the results of the weak positive sera for both viruses in order to detect the pooling effect over the sensitivity of the test. Results of pooling 20 different sera did not give good results for the antibodies to ASFV nor to CSFV detection, since weak positive signals were under the cut off established value (Data not shown).

Figure 4A shows the titration curves for the weak positive serum to ASFV spiked in the 4 and 9 negative sera, making the 5- and 10-pool, respectively. The reactivity against the three target antigens were assessed. A higher response can be observed to ASFV-antigens in all the pools when compared to the E2 antigen. The highest difference appears in the 5-pool sample, where the signal does not decrease in the first dilutions. The 10-pool sample also exhibits a good difference between target antigens (VP72 and VP30) and non-target antigens (E2). Preliminary results show that for the detection of antibodies to ASFV, pools of 5 and 10 different samples can be done maintaining good signals of weak positive samples and with no cross-reactivity between antigens.

In a similar way, the Figure 4B shows the titration curves for the weak positive serum to CSF pooled in the 4 and 9 negative sera. For the 5-pool assay, the response of the non-target antigens was over the cut off value (5000 MFI) whereas, in the case of the 10-pool sample, the difference between the E2 signal and the non-target antigens was high enough and negative signals were under the cut off value (Figure 4B). Thus, the selected conditions for the pooled assay would be a 10-pool sample diluted 1/10 in assay buffer.

## Discussion

African swine fever (ASF) and Classical swine fever (CSF) are two clinically indistinguishable diseases that cause high economic impact worldwide and, thus, both are included in the World Organization for Animal Health (OIE) list (42). In recent years, several outbreaks of both diseases have been detected in Eastern Europe, what increases the probability of encounter these two viruses in a same area (4, 13, 19, 24) what leads to the necessity of having fast and reliable tools for the differential diagnosis. In this study, a triplex assay has been optimized for the simultaneous detection of antibodies against both etiological agents, based on the xMAP Technology.

For the detection of antibodies against ASFV, both VP72 and VP30 antigens, showed a similar behavior against the experimental sera with rather good rates of sensitivity and specificity (Figures 2A,B). Three out of the four false positive results obtained with the VP72 coupled to the bead region #12 and the six obtained with the VP30 coupled to the bead region #15 (Table 2) were obtained from pigs at different days post-infection, mostly within days 10 and 15 post-infection. This observation may mean that the newly developed multiplex test is more sensitive than the ELISA used as reference techniques, being able to detect the infection at earlier times post infection. If these sera were considered positive instead of false negative samples, the newly developed assay would exhibit an increase in sensitivity and specificity values. Both antigens, as shown in Table 2, can detect the same ratio of positive and negative samples separately. However, VP30 appears to be a good antigen for ASF diagnosis, half the amount of protein is needed to reach the same results when compared to VP72 and more positive samples are detected. This observation has also been described in previous studies (5).

Taking together the reactivity of a given serum against VP72 and VP30, the values of sensitivity were slightly increased to 97.3% with a 98.3% specificity (Table 2). By the observation of these results, including both antigens in the multiplex assay seems to be the best strategy for ASF diagnosis, since it increases the sensitivity value of the assay. This could be especially of interest when analyzing field samples, where animals can react differently to viral exposure presenting diverse levels of antibodies to each of the virus proteins.

Moreover, if we consider that six out of the seven false positive samples came from animals at different days post-infection the developed test can bring an increase on the assay sensitivity, that specificity parameter would be also increased to 99.8% with a sensitivity of 97.4%. This hypothesis is strengthened by the observation in Figure 3, where the newly developed test can detect a higher percentage of positive samples in the 8–15 dpi group (from a 58% to a 68%) as well as for the 16–28 dpi group, in which the percentage is increased from 90 to 97%. When we analyzed samples after 1 month of infection, the percentage of positive samples is almost the same for both techniques. This would mean that the bead-based assay is slightly more sensitive than the reference technique used in this study, detecting infection at earlier dpi.

For detection of antibodies to CSFV, even though more positive sera from CSFV-infected animals should be analyzed to have a statistically representative value of sensitivity and specificity, a great correlation between positive and negative samples is observed, reaching a sensitivity of 95.7% and a specificity of 99.8% (Figure 2C). Moreover, the highest MFI signals observed in the negative samples were obtained from animals infected with BDV or BVDV, two *Pestivirus* related to the CSFV whose differentiation is complicated because they are highly cross-reactive antigenically (43).

By the combination of the three antigens, the developed multiplex assay shows great sensitivity and specificity parameters for the differential diagnosis of animals infected with ASFV or CSFV.

Surveillance studies are a priority when talking about high economic impact diseases such as the ones described (ASF, CSF), and it may therefore be beneficial to use pooling of samples to analyse the greatest number of animals per assay. The pooling of samples from several individuals for a single test has long been advocated as a way of reducing the cost and effort of diagnostic testing. In the veterinary field it has been used for the identification of infected individuals and populations (44, 45), and even the OIE recognize the utility of pooled samples, although it will require the determination of their own sensitivity and specificity parameters (46). Results obtained in this study indicate that pooling of 10 different sera is a good alternative to increase the high throughput screening options of the developed test, since it allows the detection of antibodies to both pathogens in the same conditions. Best conditions were established at the 1/10 dilution of the whole pool in assay buffer, which showed no cross-reactivity between target antigens and promising values of MFI for weak positive samples (Figure 4). A more in depth analysis must be done to establish the sensitivity and specificity of the assay in pooled conditions, since previous studies described an increase in specificity of pooled sera and a decrease in sensitivity when changing from unique to pooled sample analysis. This was due to the cut off readjustment for pooled samples analysis (47, 48).

The maintenance of animal health in production species and, particularly in swine, includes the control of a wide range of infectious diseases affecting both, economic and public health aspects. To date, these health evaluations are done with individual assays, and this forces the application of control plans centered in one unique pathology. The use of multiplex assays would dramatically help in those surveillance plans, by allowing the development of one unique plan for a complex infectious disease panel. Moreover, analysis of multiple analytes at once, instead of running several tests in parallel, presents several advantages compared to traditional methods, including saving labor, time and reducing user error and variability between independent assays.

It must be taken into account that this study only included positive samples experimentally obtained, in which animals were inoculated with high viral doses and trough clear inoculation routes. Real samples that reflect field conditions needs to be analyzed to determine the accuracy of the newly developed test and its diagnostic parameters.

This triplex assay would be the starting point for the development of a multiplex assay that include other diseases of special interest in swine. This multiplex assay can be of great interest and application in prevention, control and even eradication plans development.

## Data Availability

The datasets generated for this study are available on request to the corresponding author.

## Ethics Statement

The animal study was reviewed and approved by UK Home Office under the Animals (Scientific Procedure) Act UK 1986.

## Author Contributions

CA performed the experiments and drafted the manuscript. TR produced the recombinant proteins used in this study. PS and PR contributed to the design of the study and analysis, interpretation of data, and also critically revised the manuscript. SB provided CSF positive and negative experimental samples. LD provided ASF positive and negative experimental samples. All authors read and approved the final manuscript.

### Conflict of Interest Statement

The authors declare that the research was conducted in the absence of any commercial or financial relationships that could be construed as a potential conflict of interest.

## References

[1] Dixon LK Chapman DA Netherton CL Upton C. African swine fever virus replication and genomics. Virus Res. (2013) 173:3–14. 10.1016/j.virusres.2012.10.020 23142553

[2] Alejo A Matamoros T Guerra M Andres G. A proteomic atlas of the African swine fever virus particle. J Virol. (2018) 92:e01293–18. 10.1128/JVI.01293-18 30185597 PMC6232493

[3] Cubillos C Gomez-Sebastian S Moreno N Nunez MC Mulumba-Mfumu LK Quembo CJ. African swine fever virus serodiagnosis: a general review with a focus on the analyses of African serum samples. Virus Res. (2013) 173:159–67. 10.1016/j.virusres.2012.10.021 23131491

[4] Gallardo C Nieto R Soler A Pelayo V Fernández-Pinero J Markowska-Daniel I. Assessment of African swine fever diagnostic techniques as a response to the epidemic outbreaks in Eastern European Union Countries: how to improve surveillance and control programs. J Clin Microbiol. (2015) 53:2555–65. 10.1128/JCM.00857-15 26041901 PMC4508403

[5] Gimenez-Lirola LG Mur L Rivera B Mogler M Sun Y Lizano S. Detection of African swine fever virus antibodies in serum and oral fluid specimens using a recombinant protein 30 (p30) dual matrix indirect ELISA. PLoS ONE. (2016) 11:e0161230. 10.1371/journal.pone.0161230 27611939 PMC5017782

[6] Sanchez-Vizcaino JM Mur L Gomez-Villamandos JC Carrasco L. An update on the epidemiology and pathology of African swine fever. J Comp Pathol. (2015) 152:9–21. 10.1016/j.jcpa.2014.09.003 25443146

[7] Sanchez-Vizcaino JM (2006). African Swine Fever. Disease of Swine. Hoboken: Blackwell Publishing.

[8] Blome S Gabriel C Beer M. Pathogenesis of African swine fever in domestic pigs and European wild boar. Virus Res. Hoboken, NJ: Blackwell Publishing (2013) 173:122–30. 10.1016/j.virusres.2012.10.026 23137735

[9] Gallardo MC Reoyo AT Fernandez-Pinero J Iglesias I Munoz MJ Arias ML. African swine fever: a global view of the current challenge. Porcine Health Manag. (2015b) 1:21. 10.1186/s40813-015-0013-y 28405426 PMC5382474

[10] Montgomery RE On a form of swine fever ocurring in British East Africa (Kenya Colony). J Comp Pathol Therap. (1921) 34, 159–91. 10.1016/S0368-1742(21)80031-4

[11] Arias M Sánchez-Vizcaíno JM African swine fever eradication: the Spanish model. In: Zimmerman JJ Yoon K-J Morilla A editors. Trends in Emerging Viral Infections of Swine. 1st edn. Ames, IA: Iowa State University Press (2002). p. 133–9.

[12] Sánchez-Vizcaíno JM Martínez-López B Martínez-Avilés M Martins C Boinas F Vial L Scientific Review on African Swine Fever. EFSA (2009).

[13] Gogin A Gerasimov V Malogolovkin A Kolbasov D. African swine fever in the North Caucasus region and the Russian Federation in years 2007-2012. Virus Res. (2013) 173:198–203. 10.1016/j.virusres.2012.12.007 23266725

[14] (OIE) World Organisation for Animal Health (2019). World Animal Health Information Database (WAHID Interface). Available online at: http://www.oie.int/wahis_2/public/wahid.php/Diseaseinformation/Diseaseoutbreakmaps (accessed April 16, 2019).

[15] Dixon LK Sun H Roberts H. African swine fever. Antiviral Res. (2019) 165:34–41. 10.1016/j.antiviral.2019.02.018 30836106

[16] Schulz K Staubach C Blome S. African and classical swine fever: similarities, differences and epidemiological consequences. Vet Res. (2017) 48:84. 10.1186/s13567-017-0490-x 29183365 PMC5706370

[17] Konig M Lengsfeld T Pauly T Stark R Thiel HJ. Classical swine fever virus: independent induction of protective immunity by two structural glycoproteins. J Virol. (1995) 69:6479–86. 7666549 10.1128/jvi.69.10.6479-6486.1995 PMC189549

[18] Van Rijn PA Bossers A Wensvoort G Moormann RJ. Classical swine fever virus (CSFV) envelope glycoprotein E2 containing one structural antigenic unit protects pigs from lethal CSFV challenge. J Gen Virol. (1996) 77:2737–45. 10.1099/0022-1317-77-11-2737 8922467

[19] Blome S Staubach C Henke J Carlson J Beer M. Classical Swine fever-an updated review. Viruses. (2017) 9:E86. 10.3390/v9040086. 28430168 PMC5408692

[20] Hanson RP. Origin of hog cholera. J Am Vet Med Assoc. (1957) 131:211–8. 13462882

[21] Edwards S Fukusho A Lefevre PC Lipowski A Pejsak Z Roehe P. Classical swine fever: the global situation. Vet Microbiol. (2000) 73:103–19. 10.1016/S0378-1135(00)00138-3 10785321

[22] Paton DJ Greiser-Wilke I. Classical swine fever–an update. Res Vet Sci. (2003) 75:169–78. 10.1016/S0034-5288(03)00076-6 13129664

[23] Beer M Goller KV Staubach C Blome S. Genetic variability and distribution of Classical swine fever virus. Anim Health Res Rev. (2015) 16:33–9. 10.1017/S1466252315000109 26050570

[24] Moennig V. The control of classical swine fever in wild boar. Front Microbiol. (2015) 6:1211. 10.3389/fmicb.2015.01211 26594202 PMC4635204

[25] Postel A Nishi T Kameyama KI Meyer D Suckstorff O Fukai K. Reemergence of classical swine fever, Japan, 2018. Emerg Infect Dis. (2019) 25:1228–31. 10.3201/eid2506.181578. 30870139 PMC6537743

[26] Aguero M Fernandez J Romero LJ Zamora MJ Sanchez C Belak S. A highly sensitive and specific gel-based multiplex RT-PCR assay for the simultaneous and differential diagnosis of African swine fever and classical swine fever in clinical samples. Vet Res. (2004) 35:551–63. 10.1051/vetres:2004031 15369658

[27] Haines FJ Hofmann MA King DP Drew TW Crooke HR. Development and validation of a multiplex, real-time RT PCR assay for the simultaneous detection of classical and African swine fever viruses. PLoS ONE. (2013) 8:e71019. 10.1371/journal.pone.0071019 23923045 PMC3724773

[28] Sastre P Perez T Costa S Yang X Raber A Blome S. Development of a duplex lateral flow assay for simultaneous detection of antibodies against African and classical swine fever viruses. J Vet Diagn Invest. (2016) 28:543–9. 10.1177/1040638716654942 27400954

[29] Christopher-Hennings J Araujo KP Souza CJ Fang Y Lawson S Nelson EA. Opportunities for bead-based multiplex assays in veterinary diagnostic laboratories. J Vet Diagn Invest. (2013) 25:671–91. 10.1177/1040638713507256 24153036

[30] Jones LP Zheng HQ Karron RA Peret TC Tsou C Anderson LJ. Multiplex assay for detection of strain-specific antibodies against the two variable regions of the G protein of respiratory syncytial virus. Clin Diagn Lab Immunol. (2002) 9:633–8. 10.1128/cdli.9.3.633-638.2002 11986272 PMC119994

[31] Perraut R Richard V Varela ML Trape JF Guillotte M Tall A. Comparative analysis of IgG responses to Plasmodium falciparum MSP1p19 and PF13-DBL1alpha1 using ELISA and a magnetic bead-based duplex assay (MAGPIX(R)-Luminex) in a Senegalese meso-endemic community. Malar J. (2014) 13:410. 10.1186/1475-2875-13-410 25326042 PMC4221706

[32] Lawson S Lunney J Zuckermann F Osorio F Nelson E Welbon C. Development of an 8-plex Luminex assay to detect swine cytokines for vaccine development: assessment of immunity after porcine reproductive and respiratory syndrome virus (PRRSV) vaccination. Vaccine. (2010) 28:5356–64. 10.1016/j.vaccine.2010.05.016 20488263

[33] Anderson S Wakeley P Wibberley G Webster K Sawyer J. Development and evaluation of a luminex multiplex serology assay to detect antibodies to bovine herpes virus 1, parainfluenza 3 virus, bovine viral diarrhoea virus, and bovine respiratory syncytial virus, with comparison to existing ELISA detection methods. J Immunol Methods. (2011) 366:79–88. 10.1016/j.jim.2011.01.010 21277304

[34] Leblanc N Cortey M Fernandez Pinero J Gallardo C Masembe C Okurut AR. Development of a suspension microarray for the genotyping of African swine fever virus targeting the SNPs in the C-terminal end of the p72 gene region of the genome. Transbound Emerg Dis. (2013) 60:378–83. 10.1111/j.1865-1682.2012.01359.x 22776009

[35] Chen TH Lee F Lin YL Pan CH Shih CN Tseng CH. Development of a multiplex luminex assay for detecting swine antibodies to structural and nonstructural proteins of foot-and-mouth disease virus in Taiwan. J Microbiol Immunol Infect. (2016) 49:196–207. 10.1016/j.jmii.2014.05.009 25074628

[36] Hoare R Thompson KD Herath T Collet B Bron JE Adams A. Development, characterisation and application of monoclonal antibodies for the detection and quantification of infectious Salmon Anaemia virus in plasma samples using luminex bead array technology. PLoS ONE. (2016) 11:e0159155. 10.1371/journal.pone.0159155 27434377 PMC4951118

[37] Sanchez-Matamoros A Beck C Kukielka D Lecollinet S Blaise-Boisseau S Garnier A. Development of a microsphere-based immunoassay for serological detection of african horse sickness virus and comparison with other diagnostic techniques. Transbound Emerg Dis. (2016) 63:e270–7. 10.1111/tbed.12340 25693720

[38] Sanchez-Matamoros A Nieto-Pelegrin E Beck C Rivera-Arroyo B Lecollinet S Sailleau C. Development of a luminex-based DIVA assay for serological detection of african horse sickness virus in horses. Transbound Emerg Dis. (2016b) 63:353–9. 10.1111/tbed.12503 27090377

[39] Sanchez-Cordon PJ Jabbar T Berrezaie M Chapman D Reis A Sastre P. Evaluation of protection induced by immunisation of domestic pigs with deletion mutant African swine fever virus BeninDeltaMGF by different doses and routes. Vaccine. (2018) 36:707–15. 10.1016/j.vaccine.2017.12.030 29254837 PMC5783716

[40] Luo Y Li S Sun Y Qiu HJ. Classical swine fever in China: a minireview. Vet Microbiol. (2014) 172:1–6. 10.1016/j.vetmic.2014.04.004 24793098

[41] Hermanson G. T, editor. Chapter 14 - Microparticles and nanoparticles. In: Bioconjugate Techniques. San Diego, CA: Academic press (2013) 549–87.

[42] Kleiboeker SB. Swine fever: classical swine fever and African swine fever. Vet Clin North Am Food Anim Pract. (2002) 18:431–51. 10.1016/S0749-0720(02)00028-2 12442576

[43] Ridpath JF. BVDV genotypes and biotypes: practical implications for diagnosis and control. Biologicals. (2003) 31:127–31. 10.1016/S1045-1056(03)00028-9 12770544

[44] Viña M Panadero R Díaz P Fernández G Pérez A Díez-Baños P. Evaluation of the use of pooled fecal samples for the diagnosis of protostrongylid infections in sheep. Vet Parasitol. (2013) 197:231–4. 10.1016/j.vetpar.2013.05.013 23747001

[45] Hall LM Munro LA Wallace IS Mcintosh R Macneish K Murray AG. An approach to evaluating the reliability of diagnostic tests on pooled groups of infected individuals. Prev Vet Med. (2014) 116:305–12. 10.1016/j.prevetmed.2014.01.021 24534442

[46] Oie (2018). Terrestrial Animal Health Code. Office International des Épizootes.

[47] Cahoon-Young B Chandler A Livermore T Gaudino J Benjamin R. Sensitivity and specificity of pooled versus individual sera in a human immunodeficiency virus antibody prevalence study. J Clin Microbiol. (1989) 27:1893–5. 2504779 10.1128/jcm.27.8.1893-1895.1989 PMC267694

[48] Rovira A Cano JP Munoz-Zanzi C. Feasibility of pooled-sample testing for the detection of porcine reproductive and respiratory syndrome virus antibodies on serum samples by ELISA. Vet Microbiol. (2008) 130:60–8. 10.1016/j.vetmic.2007.12.016 18243590

